# Anabolic steroid consumption among gym-goers in Amman: knowledge, attitudes, and behaviors

**DOI:** 10.3389/fspor.2024.1404551

**Published:** 2024-08-29

**Authors:** Walaa AlKasasbeh, Hatem Shlool, Sajeda Alnaimat

**Affiliations:** ^1^ Department of Physical and Health Education, Faculty of Education Sciences, Al-Ahliyya Amman University, Al-salt, Jordan; ^2^ Department of Physical Education, Faculty of Sport Science, The University of Jordan, Amman, Jordan

**Keywords:** knowledge, attitudes, behaviors, hormones, gyms, anabolic-androgenic steroids, Amman

## Abstract

**Background:**

The use of Anabolic-androgenic steroids (AAS) among gym members has become a significant concern due to their impact on physical training and performance. Research worldwide indicates a notable prevalence of AAS use among athletes and gym attendees, often involving substances that are neither safe nor legal.

**Objectives:**

This study aims to determine the prevalence of AAS use among gym attendees in Amman, Jordan, and to explore the knowledge, attitudes, and behaviors associated with AAS use.

**Methods:**

The study involved 399 participants from 35 randomly selected gyms in the metropolitan area of Amman, Jordan. A cluster sampling technique was used to select a diverse and representative sample of gym attendees. Data was collected using a self-administered questionnaire that assessed AAS use, knowledge, attitudes, and behavioral factors. Statistical analyses were conducted using chi-square tests to explore the relationships between AAS use and categorical variables, while logistic regression was employed to identify predictors of AAS use.

**Results:**

The analysis revealed significant associations between AAS use and various factors, including knowledge, attitudes, behavioral factors, and demographic variables such as gender, age, exercise frequency, reasons for exercise, and total exercise duration. The study identified key predictors of AAS use among gym attendees in Amman, highlighting the importance of demographic and behavioral factors.

**Conclusion:**

The findings underscore the need for targeted interventions to address misconceptions and promote safer practices among gym-goers in Amman. The study provides critical insights that can guide the development of strategies, policy adjustments, and educational initiatives aimed at reducing AAS misuse and fostering a healthier gym culture in the region.

## Introduction

Anabolic-androgenic steroids (AAS), comprising testosterone and its related compounds metabolically altered variants, are frequently employed to augment stimulate protein synthesis, foster muscle growth, and boost erythropoiesis (1). Prominent formulations like nandrolone and trenbolone can be found in injectable or oral formats (2). These compounds have been influential since the 1940s in rehabilitating individuals recovering from injuries such as burns, trauma, and surgical procedures (3). Extensive research has expanded their applications to include the treatment of aging-related conditions like hypogonadism, osteoporosis, and diseases such as cachexia resulting from HIV and cancer (4). Elevated doses contribute to the enhancement of protein synthesis and the preservation of lean mass (5, 6). Research indicates potential applications in leukemia treatment through the stimulation of bone marrow proliferation and hematopoiesis (7). Furthermore, androgens can stimulate growth hormone synthesis, addressing conditions causing Diminished height and inhibited growth, such as turner syndrome. Continuing medical research indicates the potential use of testosterone (T) or testosterone in conjunction with the drug anastrozole (A) in breast cancer treatment. A lower incidence of breast cancer is observed in women with hormone deficiency when administered sufficient doses of these treatments. Implants combining T and A placed around malignant tumors result in a notable decrease in tumor size, indicating a direct protective and antiproliferative impact (8).

According to health organizations (9), AAS show many side effects, including cardiac disorders and metabolic disturbances (10). Reports have indicated phenomena such as oxidative stress, vascular dysfunction disorders among AAS users, along with inhibition of vascular formation and increased sympathetic nervous activity, according to the reports (11, 12). All these phenomena contribute to elevated blood pressure (13). Studies indicate that users of AAS not only have higher blood pressure levels compared to those in the control group, but these values also align with those diagnosed in patients with hypertension, despite being at a young age and without accompanying diseases (14, 15).

The use of AAS among gym-goers is increasingly popular (16) with individuals seeking treatments to build muscle, reduce fat, and improve overall fitness (17). The goal is to aid the body's hormone production for fitness objectives, including increased energy levels and improved recovery time from workouts (18–20). While some may be concerned about the safety of hormone use, numerous studies have demonstrated their safety and effectiveness when used as instructed (21, 22). However, misuse of hormones is prevalent, often without seeking medical advice, leading to unhealthy practices and potential long-term consequences (23, 24). Excessive hormone abuse can result in serious side effects such as dehydration, dizziness, and, in some cases, death (25, 26). Notably, many steroids available over the counter are not regulated, posing risks with hidden ingredients or toxic elements (27, 28).

While AAS are known to enhance various aspects, including athletic performance, muscle building, appearance, sexual desire, and self-assurance (29), their abusive use has progressively revealed detrimental effects. Prolonged and excessive usage can disrupt secretion of endogenous hormones, resulting in either reversible or irreversible harm. Common side effects encompass increased sebaceous gland activity, resulting in skin conditions such as the occurrence of acne vulgaris and excessive hair growth (30).

Athletes engaging in the abuse of AAS face a heightened risk of tendon rupture, which can significantly impede their athletic careers. Prolonged use may contribute to psych behavioral disorders, manifesting as headaches, irritability, depression, and, in extreme cases, anabolic-androgenic steroid dependence syndrome, occasionally leading to violence and suicide (31, 32). Excessive oral intake of AAS burdens the hepatic-renal system, exacerbating liver damage and contributing to liver or kidney diseases, including impaired coagulation, cirrhosis, renal enlargement, and renal failure (33, 34). Moreover, steroid abuse elevates the risk of cardiovascular diseases, posing a serious threat to user safety (30, 35). It is crucial for individuals to be aware of these potential health risks associated with the misuse of AAS and seek professional guidance to mitigate adverse effects.

The World Anti-Doping Agency (WADA) has published its prohibited list for 2024, which includes a wide range of substances and methods banned for use by athletes both in and out of competition (36). This list includes substances such as Anabolic Androgenic Steroids (AAS), which encompass testosterone, nandrolone, stanozolol, and trenbolone. Additionally, prohibited substances include peptide hormones, growth factors, and erythropoiesis-stimulating agents like erythropoietin (EPO) and its mimetics (36). The list also prohibits substances such as clenbuterol, selective androgen receptor modulators (SARMs), and certain drugs like tramadol, which will be banned in competition starting January 2024 due to their potential for misuse and performance enhancement (36).

Studies conducted on Jordanian society and elsewhere indicate a growing phenomenon of taking steroids, especially anabolic steroids, among young people and athletes (37, 38) indicated that 19% of gym-goers take anabolic steroids, with testosterone being the most common steroid among this demographic. Given the danger of steroid abuse in general and anabolic steroids in particular due to their effects on health and psychological aspects (31, 32), researchers have concluded that there is relatively little information about knowledge, attitudes, and behaviors of Anabolic-androgenic steroid and their impact on the health, and psychological aspects of steroid users in Jordan. The current study has a central goal of assessing the level of knowledge and awareness among gym-goers regarding the potential health risks associated with the misuse or incorrect use of AAS. This gathered information will serve as a valuable resource in directing efforts toward addressing the issue of Anabolic-androgenic steroid abuse. Moreover, it will contribute to the formulation of informed strategies and the modification of existing laws, with a specific focus on the healthcare system's perspective.

In alignment with this, the primary objective of the research is to delve into the knowledge, attitudes, and behaviors prevalent among individuals who regularly attend gyms in Amman, Jordan, concerning the use of AAS. The investigation seeks to provide a comprehensive understanding of the factors influencing Anabolic-androgenic steroid use in this demographic and pave the way for targeted interventions and policy adjustments aimed at promoting health and well-being.

In line with this objective, the study hypothesizes that there exists a significant difference in the levels of knowledge, attitudes, and behaviors related to AAS and their effects among individuals who attend gyms. The investigation seeks to uncover nuanced insights into the factors influencing AAS use within this demographic, providing a foundation for targeted interventions and policy adjustments aimed at promoting the overall health and well-being of gym-goers in Amman. Additionally, the study posits that there is a significant association between the levels of knowledge, attitudes, and behaviors regarding AAS and their effects and the likelihood of engaging in AAS use among individuals exercising at gyms in Amman. By exploring these relationships, the research aims to contribute valuable insights into the complex interplay of factors influencing Anabolic-androgenic steroid use, paving the way for evidence-based interventions to mitigate potential risks and enhance the overall health outcomes for gym-goers.

## Methods and materials

### Participants

The study involved 399 gym users in Amman, Jordan, recruited from 35 randomly selected gyms in the metropolitan area. The participants were selected using a cluster sampling technique to ensure diversity in representation. Individuals were approached on randomly selected days with an expected average of 12 individuals per gym. Informed consent was obtained from each participant. To be eligible, participants had to be 18 years or older, regular gym users for at least three months, and not staff members at the fitness centers.

Participants, comprising 165 females (41.3%) and 234 males (58.6%), were aged 18–35 years, with 38.3% aged 18–25, 26.1% aged 26–30, and 22.3% aged 31–35. Exercise frequency varied, with 21.8% exercising less than 3 times per week, 53.4% exercising 3–5 times, and 24.8% exercising more than 5 times. Most participants (79.9%) had been exercising for more than a year. Gym usage reasons included 26.8% for other reasons, 25.8% for competitions, and 47.4% to improve body appearance.

### Procedure

The researchers approached individuals attending the selected gyms, explained the study, and obtained informed consent. The data collection was done through a self-administered questionnaire. The researchers made themselves accessible throughout the study to provide support and address any concerns. The study specifically targeted regular gym users, and data collection was carried out on randomly selected days. To exclude minors, participants had to be 18 years or older. Staff members at the fitness centers were excluded from the study.

### Instruments

The primary instrument employed for data collection in this study was a questionnaire, which had been pre-established for the specific research objectives (39), Only the section pertaining to AAS was taken from this questionnaire. The translation of the questionnaire from English to Arabic was rigorously validated to ensure its appropriateness for the local context. The validation process involved eight experts in fields such as assessment, measurements, Arabic language, specialized English language, and nutrition. This thorough validation process ensured that the translated questionnaire was accurate, culturally relevant, and reliable for data collection in Amman, Jordan. The questionnaire comprised five sections: the first focused on gathering sociodemographic information, encompassing details such as age, sex, frequency of weekly exercise, total exercise duration, and reasons for engaging in physical activity. The second section delved into the usage of AAS, exploring whether participants utilized these substances, the specific types employed, and the sources from which they obtained them. The third section targeted participants’ knowledge about AAS. Attitudes were assessed in the fourth section using a three-point Likert scale, providing insights into respondents’ agreement, uncertainty, or disagreement regarding relevant topics. The final section encompassed twenty questions related to behaviors, utilizing a four-degree scale to rate responses (yes, often, sometimes, never).To refine the questionnaire and assess its practicality, a pilot study involving 60 participants was conducted prior to the main investigation, focusing on logistical considerations and data collection procedures.

### Ethical considerations

The study (Approval number: FES-18G-115) received approval from the Ethics Committee of Al-Ahliyya Amman University. Verbal permission was obtained from gym administrators to monitor survey distribution and collection and to address any concerns participants might have had. each participant provided informed consent before being included in the study.

### Statistical analysis

The study employed statistical methods to analyze the data and examine relationships between different variables. Firstly, the Chi-square test was used to assess the statistical relationships between the use of AAS and other categorical variables such as sex, age, weekly exercise frequency, reason for engaging in sports, and duration of exercise. Additionally, the Chi-square test was used to analyze relationships between the level of knowledge about AAS, individuals’ attitudes, and behaviors related to AAS use. Finally, descriptive statistics were utilized to summarize social data, including the distribution of sex, age, and weekly exercise, encompassing total counts and percentages. These statistical methods were employed to investigate statistically significant differences or associations between various groups of study participants, providing statistical insights into the relationships between different variables. IBM SPSS version 20 for Windows operating system was used for statistical analysis.

## Results

Table 1 offers a thorough summary of the socio-demographic traits of the study participants, with a total sample size of 399 individuals. The distribution of participants by sex indicates a relatively balanced representation, with 165 females (41.3%) and 243 males (58.6%). In terms of age groups, the majority falls within the 18–25 years bracket (38.3%), followed by 26–30 years (26.1%), and 31–35 years (22.3%). The frequency of exercise per week reveals that a significant portion of participants engage in physical activity regularly, with 53.4% exercising 3–5 times a week, 24.8% more than 5 times, and 21.8% less than 3 times. Participants reported varied motivations for using the gym, with 47.4% aiming to improve their body appearance, 25.8% participating in competitions, and 26.8% citing other reasons. Concerning the total period of exercise, the majority of participants (79.9%) reported engaging in physical activity for more than a year, while 20.1% had a total exercise duration of less than one year.

**Table 1:** Socio-demographic characteristics of study participants (*N* = 399).

Socio-demographic *N* (%)			
Sex	Female	Male	
	165 (41.3)	243 (58.6)	
Age	18–25 years	26–30 years	31–35 years
	153 (38.3)	104 (26.1	89 (22.3)
Frequency of exercise per week	<3 times	3–5 times	>5 times
	87 (21.8)	213 (53.4)	99 (24.8)
Reason for using the gym	Other reasons	Participate in competitions	Improve the appearance of the body
	107 (26.8)	103 (25.8)	189 (47.4)
Total period of exercise	less than one year	More than a year	
	80 (20.1)	319 (79.9)	

Table 2 presents a comprehensive analysis of the associations between various study variables and the use of AAS among individuals who engage in exercise at gyms. The data, consisting of frequencies and percentages, reveals significant patterns across different demographic and behavioral categories.

**Table 2:** The associations between study variables and AAS use among people exercising at gyms.

Use of anabolic-androgenic steroids			
Study variables	Yes *N* (%)	No *N* (%)	*p*-value
Sex			
Female	3 (1.8)	162 (98.2)	*p* = <0.001
Male	77 (32.9)	157 (67.1)	
Total	80 (20.1)	319 (79.9)	
Age			
18–25 years	25 (16.30)	128 (83.70)	*p* = <0.002
26–30 years	21 (20.20)	83 (79.80)	
31–35 years	22 (24.70)	67 (75.30)	
36 years and over	12 (22.60)	41 (77.40)	
Total	80 (20.1)	319 (79.9)	
Frequency of exercise per week			
<3 times	3 (3.4)	84 (96.6)	*p* = 0.067
3–5 times	36 (16.9)	177 (83.1)	
>5 times	41 (41.4)	58 (58.6)	
Total	80 (20.1)	319 (79.9)	
Reason for doing the exercise			
Other reasons	6 (5.6)	101 (94.4)	*p* = <0.001
Participate in competitions	59 (57.3)	44 (42.7)	
Improve the appearance of the body	15 (7.9)	174 (92.1)	
Total	80 (70.8)	319 (29.2)	
Total period of exercise			
Less than one year	31 (38.75)	49 (61.2)	*p* = <0.001
More than year	197 (61.7)	122 (38.2)	
Total	228 (20.1)	171 (79.9)	

**Table 3:** Association between KAB and use of AAS.

Knowledge, Attitudes, Behaviors (KAB) of use AAS	Agree	Unsure	Disagree	*p*-value	
Knowledge (K)	*N* (%)	*N* (%)	*N* (%)		
Anabolic steroids are food supplements	34 (8.50)	101 (25.30)	264 (66.20)	*P* = 0.037	
Anabolic steroids are AAS	278 (69.70)	97 (24.30)	24 (6.00)		
A proper nutrition includes AAS	88 (22.10)	89 (22.30)	222 (55.60)		
AAS are food supplements	67 (16.80)	101 (25.30)	231 (57.90)		
AAS are drugs	144 (36.10)	111 (27.80)	144 (36.10)		
AAS have to be prescribed only by the doctor	221 (55.40)	72 (18.00)	106 (26.60)		
Attitudes (A)	Agree	Unsure	Disagree	*p*-value	
Bodybuilding improves the health	304 (76.10)	39 (9.70)	56 (14.03)	*P* ≤ 0.001	
Bodybuilding improves the physical appearance	375 (93.98)	9 (2.25%)	15 (3.75%)		
People who practice body building need a specific diet in comparison to those who do not practice it	375 (93.98)	9 (2.25)	15 (3.75)		
For a bodybuilder a specific diet balances the highest energy consumption (calories)	376 (94)	9 (2.25)	15 (3.75)		
It is necessary to consult the doctor before taking AAS	360 (90.22)	24 (6.01)	15 (3.75)		
It is safe to buy AAS on websites	120 (30)	40 (10)	240 (60)		
To improve sport performance, you take hazardous substances for the health	49 (12.28)	12 (3.00)	338 (84.71)		
To improve sport performance, you recommend others to use AAS	43 (10.77)	60 (15.03)	296 (74.18)		
Behaviors (B)	Yes	Often	Sometimes	Never	*p*-value
In order to compensate for the most energy expenditure and/or improve sports performance, you follow a specific diet	207 (51.87)	89 (22.30)	88 (22.05)	15 (3.75)	*P* ≤ 0.001
You take AAS to offset the increased energy consumption and/or improve your sporting performance	68 (17.04)	30 (7.51)	58 (14.53)	262 (65.66)	
You ever exceeded recommended doses of AAS to offset the increased energy consumption and/or improve sports performance	24 (6.01)	19 (4.76)	30 (7.51)	326 (81.70)	
You advice to others the use of AAS	21 (5.26)	18 (4.51)	93 (23.30)	267 (66.91)	
You buy AAS on the Internet	16 (4.01)	9 (2.25)	22 (5.51)	352 (88.22)	
You buy AAS in the pharmacies	38 (9.52)	27 (6.76)	64 (16.04)	270 (67.66)	
You inform yourself at the gym about the use and type of AAS to take	118 (29.57)	44 (11.02)	95 (23.80)	142 (35.58)	
you inform yourself on the Internet about the use and type of AAS to take	125 (31.32)	58 (14.53)	107 (26.81)	109 (27.31)	
Tell your doctor/nutritionist about the use and type of AAS to be taken	135 (33.83)	37 (9.27)	59 (14.78)	168 (42.10)	
You update on the laws regulating the use and type of AAS	120 (30.07)	56 (14.03)	83 (20.80)	140 (35.08)	

Sex emerges as a critical factor, demonstrating a statistically significant association with steroid use (*p*-value ≤ 0.001). Only 1.8% of females reported using steroids, whereas a substantial 32.9% of males acknowledged such usage.

Age also plays a role in steroid prevalence, with statistically significant variations observed among different age groups (*p*-value ≤ 0.002). The highest percentage of steroid use (24.7%) is noted in the 31–35 years age category.

While not statistically significant (*p*-value = 0.067), the frequency of exercise per week shows noteworthy differences in steroid use. Those exercising more than 5 times a week exhibit the highest percentage of steroid use (41.4%).

Significant associations between the reason for exercising and steroid use are evident (*p*-value ≤ 0.001). Participants engaged in competitions display a notably higher percentage (57.3%) of steroid use compared to those exercising for other reasons or to improve body appearance.

The total period of exercise demonstrates a statistically significant association with steroid use (*p*-value ≤ 0.001). Individuals with less than one year of exercise experience a higher percentage (38.4%) of steroid use compared to those with over a year of exercise.

The presented Table 3 offers a comprehensive analysis of the interplay between Knowledge, Attitudes, and Behaviors (KAB) concerning the use of AAS among respondents. In the Knowledge section, respondents’ beliefs regarding anabolic steroids’ classification as either food supplements or AAS are detailed, alongside their perceptions of AAS role in nutrition. The statistical significance, as indicated by *p*-values of 0.037, underscores the importance of these beliefs. Moving to the Attitudes section, the table delves into respondents’ perspectives on bodybuilding's impact on health, the necessity of specific diets for bodybuilders, and the importance of consulting doctors before AAS use. The highly significant *p*-values of <0.001 highlight the robust connection between bodybuilding and perceived health improvement. In the Behaviors section, respondents’ reported actions related to AAS use, including dietary habits and recommendations to others, are detailed. The consistently low *p*-values of <0.001 emphasize the strong statistical associations between reported behaviors and the underlying attitudes and beliefs of respondents. Overall, these findings provide valuable insights for targeted interventions and education to address misconceptions and promote safer practices in AAS use.

## Discussion

The present study explores the knowledge, attitudes, and behaviors related to anabolic steroid use among gym-goers in Jordan, alongside their demographic variables. Despite the lower percentage of non-users compared to users in the sample, a significant 20.1% reported steroid use, aligning with global prevalence rates. Notably, this rate surpasses meta-analysis, which reported an overall prevalence of 3.3% (40), and even exceeds the range of 0.4%–35% in AAS use (41). This indicates a notably high prevalence of steroid use among gym-goers in Jordan compared to global rates, suggesting its widespread occurrence within this demographic.

The study reaffirmed the well-established relationship between sex and steroid use, with a statistically significant difference observed in the sex variable (*p* ≤ 0.001). The findings revealed that males exhibit a higher prevalence of steroid use compared to females, consistent with the results of numerous studies. For instance, a meta-analysis reported a higher prevalence rate among males (6.4%) compared to females (1.6%) (40), Additionally, the study highlighted a notable sex disparity in lifetime steroid use, with rates of 3.6% for males and 0.6% for females. These results align with the broader body of research indicating a significant sex gap in anabolic steroid use, as evidenced by studies (42–44). Researchers have attributed this gender disparity to women's generally more conservative attitudes towards steroid use compared to men (45–47). Such attitudes may stem from societal norms and expectations surrounding sex roles and appearance ideals, where men may feel greater pressure to attain muscularity and physical dominance.The observed sex disparity in steroid use can be attributed to various societal factors and cultural norms. Firstly, societal expectations regarding physical appearance play a significant role. Research suggests that society expects women to maintain a less muscular appearance compared to men (48–50). This expectation may influence women to be less inclined towards steroid use, as it contradicts societal norms surrounding femininity and beauty standards. Furthermore, studies indicate that women tend to create fewer social connections in gym settings compared to men (51). Consequently, they may have limited involvement in discussions and discourse surrounding topics like steroid use. This reduced engagement could contribute to lower rates of steroid use among women.Another contributing factor is the divergence in beauty ideals between sex. While the societal beauty ideal for women often revolves around a thin body, men are often expected to adhere to a muscular physique (48, 52). This discrepancy in beauty ideals may lead to greater societal pressure on men to achieve muscularity, potentially driving higher rates of steroid use among males. Moreover, Kanayama et al. argues that both men and women have faced social and cultural pressures to conform to prominent body ideals for decades (53). However, due to the historical emphasis on muscularity as the male ideal, there appears to be a greater prevalence of anabolic steroid use among men. Societal expectations regarding physical appearance, sex -specific beauty ideals, and cultural pressures contribute to the observed sex differences in steroid use.

The current study sheds light on the significant role of age in determining the prevalence of steroid use among gym-goers, with statistically significant differences observed between various age groups (*p*-value = 0.002). These findings corroborate existing research, which suggests that age plays a pivotal role in the use of anabolic steroids. Studies have highlighted the positive correlation between age and steroid use, indicating that steroid use is widespread across different age groups, particularly among athletes frequenting gyms (54, 55). For instance study in South Wales revealed that a staggering 70% of recreational gym users reported using androgenic anabolic steroids (AAS) (56). Similarly, Leifman et al. reported a high prevalence of AAS use among gym-goers, particularly among young men (57). Additionally, evidence of steroid use has been found among high school and middle school athletes, with a higher prevalence observed among male athletes (42). These collective findings underscore the notion that steroid use transcends specific age demographics but is particularly prominent among young male athletes.In line with the current study's findings, the highest rate of steroid use was noted among individuals aged 31–35 years, reaching 24.7% (58). This finding is consistent with previous research, which reported an average age of 33.6 years among steroid users (56). Moreover, it has been found that more than half of lifelong anabolic steroid users are over 25 years of age, further emphasizing the prevalence of steroid use among older individuals (54). Furthermore, these findings are reinforced by the fact that more than half of lifelong anabolic steroid users are over 25 years of age (54). This demographic trend aligns with the notion that individuals may feel pressure to maintain fitness and body appearance as they age, especially considering the decline in skeletal muscle mass and exercise capacity post-30 years, potentially driving the uptake of steroids (59).

The typical user of anabolic steroids is commonly described as a male between the ages of 20 and 40 who engages in weightlifting activities (58). Some studies, suggest that steroid use often begins at an early age (30, 60). This finding resonates with the study conducted by Leite et al., which identified young adults between 20 and 29 years old as the largest users of AAS (61). One plausible explanation for the prevalence of steroid use among younger individuals is the pressure to maintain a certain level of fitness and physical appearance. As individuals age, there is a natural decline in skeletal muscle mass and exercise capacity, particularly after the age of 30. This decline is often associated with an increase in the production of reactive oxygen species (ROS) during exercise (59). Furthermore, the accumulation of oxidized proteins may contribute to the loss of structural integrity and physiological function, potentially motivating individuals to turn to steroids as a means of counteracting these effects (62).

the combination of societal pressures to maintain physical appearance, coupled with the natural physiological changes associated with aging, may drive younger individuals, particularly those engaged in weightlifting activities, to initiate steroid use as a perceived solution to enhance performance and muscle growth.

The study revealed interesting insights into the relationship between exercise frequency, commitment to gym attendance, and steroid use among gym-goers. While the number of training times per week did not show statistical significance (*P* value = 0.067), there was a notable finding regarding the frequency of training per week. Although there were no significant differences in steroid use based on training frequency, individuals who exercised more than 5 times a week exhibited the highest rate of steroid use, accounting for 41.4% of steroid users.

Moreover, the study demonstrated a significant association between long-term commitment to exercising in gyms and steroid use, with individuals committed to gym attendance for more than a year showing a statistically significant preference for using steroids (*p* = 0.000) compared to those with shorter commitments. These findings align with existing research indicating the widespread use of AAS among gym-goers, particularly among those with consistent and prolonged engagement in training activities (41, 57).

The motivation behind steroid use among dedicated gym attendees often stems from the desire to enhance physical performance and improve body composition (63). Long-term supplementation with AAS has been associated with various physiological effects, including an increase in lean leg mass, muscle fiber size, and muscle strength, albeit dependent on dosage (64). These findings suggest that individuals may turn to steroids as a means to accelerate their fitness goals and achieve desired outcomes more rapidly.

The study findings underscore the significant impact of motivations behind sports activity on the use of anabolic steroids among gym-goers. The results revealed statistically significant differences (*p* = 0.000) in the reasons for practicing sports, with a notable percentage (57.3%) of individuals engaging in sports activities primarily to participate in competitions. This emphasis on competitive participation suggests a prevalent trend among athletes in gyms, particularly in excelling in bodybuilding competitions (65, 66). Motivations for steroid use in this context extend beyond mere competition. Individuals may seek to enhance their physical appearance and achieve desired body image ideals (67). The allure of winning competitions and gaining a competitive edge may overshadow concerns about potential health risks associated with steroid use (68). Despite these motivations, the study reveals a significant relationship between knowledge, attitudes, and steroid use.

Interestingly, the study found that greater knowledge about steroids and negative attitudes towards their use were associated with a reduction in steroid use among the study sample. This finding aligns with the notion that increasing knowledge and fostering positive attitudes can promote healthier behaviors and discourage steroid misuse (69). However, it is essential to acknowledge the complexity of these relationships (70, 71), Despite the recognition of severe neurological consequences associated with steroid abuse, mere knowledge may not suffice to prevent harmful behaviors (70).

The relationship between knowledge, attitudes, and the use of anabolic steroids has been extensively studied, revealing important insights into factors influencing steroid use behaviors. Studies have shown that individuals with greater knowledge about stimulants and more positive attitudes towards their use are more likely to engage in steroid use (72). Similarly, research demonstrated the impact of mass communication on attitudes, with exposure to anti-drug messages correlating with more negative attitudes towards steroid use (73).

Furthermore, the crucial role of knowledge in influencing steroid use behaviors, particularly among bodybuilders who demonstrated a high level of awareness about the effects of steroids (74). Collectively, these findings suggest that interventions targeting knowledge and attitudes can be effective in reducing steroid use by influencing individuals’ perceptions and behaviors. By increasing awareness about the risks and consequences associated with steroid use and fostering negative attitudes towards its use, interventions can help deter individuals from engaging in steroid misuse. Educational campaigns, mass media messages, and targeted interventions within gym settings can play a significant role in disseminating accurate information and promoting healthier attitudes towards steroid use (75, 76).

The findings of this study highlight a specific demographic profile associated with a higher likelihood of steroid use despite knowledge and negative attitudes toward its use. Males aged 31–35 who engage in intense training regimens of more than 5 times a week for over a year and participate in sports competitions are identified as particularly vulnerable to steroid use. Interestingly, these results suggest that knowledge and attitudes alone may not be sufficient to deter steroid use behavior (77). This underscores the complexity of factors influencing steroid use behaviors, necessitating consideration of additional variables such as beliefs and social influences (78). Research emphasizes the significant role of social influences, including peer pressure and societal norms, in motivating steroid use among certain demographic groups, such as white adolescent males (79–81). Additionally, the impact of social support networks and perceptions of the relative safety of steroids in influencing use behaviors (82). Moreover, the significance of various personal and health-related factors, such as immigrant status, self-esteem, and prescription drug use, in predicting steroid use (55). These studies emphasize the multifaceted nature of steroid use behaviors, indicating that a range of factors beyond knowledge and attitudes contribute to individuals’ decisions to use steroids. By considering beliefs, social influences, and personal characteristics, interventions can be tailored to address the diverse array of factors influencing steroid use behaviors effectively. Understanding the interplay between these factors is crucial for developing comprehensive strategies aimed at preventing steroid misuse and promoting healthier behaviors among vulnerable populations.

The researchers observed a surprising result contrary to their initial hypothesis regarding the relationship between knowledge, attitudes, and the use of steroids. Despite expectations, the study findings revealed no significant correlation between the level of knowledge about steroids and attitudes toward their use, nor did it correlate with actual steroid use behaviors. This unexpected outcome suggests that simply increasing knowledge about steroids may not necessarily influence individuals’ attitudes or behaviors related to steroid use. Furthermore, the study delved into gym-goers’ behaviors regarding their knowledge and understanding of steroid use. The results revealed a concerning lack of awareness among participants regarding the sources from which they obtain information about AAS and the legal aspects regulating their use. A significant portion of respondents relied on the Internet (31.32%) for information, indicating a potential for misinformation or biased sources. Additionally, a notable percentage (42.10%) did not disclose their AAS usage to healthcare professionals such as doctors or nutritionists, potentially missing out on crucial guidance or oversight. Moreover, a substantial portion of participants (35.08%) admitted to not updating themselves on the laws regulating AAS use, highlighting a lack of awareness regarding legal implications and safety guidelines. Furthermore, misconceptions were evident, with over a third of respondents (36.10%) erroneously believing that AAS are not stimulants, which could contribute to misconceptions about their effects and risks. Additionally, a concerning percentage (26.60%) indicated that consulting a doctor before AAS use was unnecessary, potentially neglecting important health considerations and risking adverse effects.

These findings underscore the importance of targeted educational interventions aimed at enhancing awareness among gym-goers about the sources of reliable information on AAS and the legal aspects governing their use. Additionally, efforts should be made to promote the importance of consulting healthcare professionals, such as doctors or nutritionists, before initiating AAS supplementation regimens. By addressing these knowledge gaps and misconceptions, interventions can help mitigate the risks associated with AAS use and promote safer and more informed practices among gym-goers.

### Limitations of study

The study, conducted in Amman, Jordan, has several limitations that may affect the generalizability and interpretation of its findings. Firstly, the cultural, social, and economic context of Amman might limit the generalizability of the findings to other regions or countries with different characteristics. This implies that the patterns and behaviors observed in this study may not be applicable to gym-goers in areas with different social norms, economic conditions, or fitness cultures. Consequently, the interventions designed based on this study might need significant adjustments to be effective in other contexts, reducing the broad applicability of the study's recommendations. Secondly, ethical and legal concerns surrounding AAS use may have led to underreporting, as participants might have been hesitant to disclose their AAS use due to potential legal implications or the stigma associated with steroid use. This underreporting could result in an underestimation of the actual prevalence of AAS use, skewing the data and potentially minimizing the perceived scope of the issue. As a result, the study's findings might not fully represent the true extent of AAS use among gym-goers in Amman, which could affect the accuracy and effectiveness of any interventions developed based on these findings. Additionally, interviewer bias could have influenced participants’ responses, as the presence of researchers during the questionnaire administration might have impacted how participants answered. If participants felt judged or uncomfortable, they might have altered their responses to align with perceived social desirability, leading to biased data. This influence can compromise the authenticity of the reported behaviors and attitudes, thus affecting the reliability of the study's conclusions and the credibility of its recommendations for addressing AAS use.

Non-response bias is another limitation, as individuals who chose not to participate in the study might have different characteristics or behaviors compared to those who did participate. If non-respondents are systematically different in their AAS use or related attitudes and behaviors, the study's findings may not accurately reflect the wider gym-going population. This bias could result in an incomplete or distorted picture of AAS use patterns, thereby limiting the comprehensiveness of the study's insights and potentially leading to ineffective or misdirected interventions. Lastly, while the sample size was balanced in terms of gender, the statistical power to detect smaller effects might be limited. The relatively small sample size could restrict the ability to identify subtle yet important relationships between variables, reducing the robustness of the findings. This limitation means that the study may have missed identifying some relevant factors influencing AAS use, which could result in less comprehensive intervention strategies. Addressing these limitations in future research could provide a more robust understanding of AAS use among gym-goers in Amman and beyond. Enhanced methodologies, larger and more diverse samples, and strategies to mitigate bias would contribute to a deeper and more accurate comprehension of the factors influencing AAS use, thereby supporting the development of more effective and targeted interventions. This, in turn, would enhance the reliability and applicability of the findings, leading to better-informed strategies and policies for curbing AAS misuse and promoting healthier gym cultures.

## Conclusion

In conclusion, this comprehensive study delving into the prevalent use of AAS among gym attendees in Amman, Jordan. The findings underscore significant associations with various demographic factors, including sex, age, exercise frequency, reasons for exercise, and total exercise duration. Particularly noteworthy is the higher prevalence of AAS use among males and individuals aged 31–35 years, shedding light on demographic groups that may be more susceptible to engaging in such practices. This demographic specificity provides a targeted focus for interventions, allowing for more effective strategies to address misconceptions and promote safer practices (83).

The study strongly advocates for targeted interventions, emphasizing the urgency of addressing misconceptions and fostering a culture of safe and informed AAS use. This emphasis on targeted interventions aligns with the overarching goal of contributing to the overall health and well-being of gym-goers in the region, highlighting the importance of responsible practices in pursuit of fitness objectives. Moreover, the identified patterns and correlations serve as a foundation for informed strategies, policy adjustments, and educational initiatives. By acknowledging and understanding the factors influencing AAS use, authorities and healthcare professionals can tailor interventions to effectively mitigate potential risks and enhance the overall health outcomes for this demographic.

In the ongoing discourse on AAS use, this research stands as a significant contribution, advocating for evidence-based interventions (84) that not only curb the misuse of AAS but also work towards fostering a healthier gym culture in Amman. The study serves as a call to action, urging stakeholders to collaborate in implementing effective measures that prioritize the well-being of gym-goers and promote sustainable and safe fitness practices.

## Data Availability

The raw data supporting the conclusions of this article will be made available by the authors, without undue reservation.
