# Corrigendum: Anabolic steroid consumption among gym-goers in Amman: knowledge, attitudes, and behaviors

**DOI:** 10.3389/fspor.2024.1521478

**Published:** 2024-11-27

**Authors:** Walaa AlKasasbeh, Hatem Shlool, Sajeda Alnaimat

**Affiliations:** ^1^ Department of Physical and Health Education, Faculty of Education Sciences, Al-Ahliyya Amman University, Al-salt, Jordan; ^2^ Department of Physical Education, Faculty of Sport Science, The University of Jordan, Amman, Jordan

**Keywords:** knowledge, attitudes, behaviors, hormones, gyms, anabolic-androgenic steroids, Amman

A Corrigendum on Anabolic steroid consumption among gym-goers in Amman: knowledge, attitudes, and behaviors By AlKasasbeh W, Shlool H, Alnaimat S. (2024) Front Sports Act Living. 6:1404551. doi: 10.3389/fspor.2024.1404551

Incorrect Affiliation:

In the published article, there was an error in affiliation(s) [Walaa AlKasasbeh^1,2^]. Instead of “(Walaa AlKasasbeh^1,2^)”, it should be (Walaa Alkasasbeh^1^).

Please delete (^2^Program of Sports Management and Training, Department of Administration and Curriculum, Faculty of Arts and Educational Sciences, Middle East University, Amman,).

Correct Affiliation it is:

(Walaa AlKasasbeh^1^*, Hatem Shlool^1^ and Sajeda Alnaimat^2^)

(^1^Department of Physical and Health Education, Faculty of Education Sciences, Al-Ahliyya Amman University, Al-salt, Jordan, ^2^Department of Physical Education, Faculty of Sport Science, The University of Jordan, Amman, Jordan)

The authors apologize for this error and state that this does not change the scientific conclusions of the article in any way. The original article has been updated.

